# IROA: the International Register of Open Abdomen. An international effort to better understand the open abdomen: call for participants

**DOI:** 10.1186/s13017-015-0029-2

**Published:** 2015-08-16

**Authors:** Federico Coccolini, Fausto Catena, Giulia Montori, Marco Ceresoli, Roberto Manfredi, Gabriela Elisa Nita, Ernest E. Moore, Walter Biffl, Rao Ivatury, James Whelan, Gustavo Fraga, Ari Leppaniemi, Massimo Sartelli, Salomone Di Saverio, Luca Ansaloni

**Affiliations:** General Emergency and Trauma Surgery, Papa Giovanni XXIII Hospital, Piazza OMS 1, 24127 Bergamo, Italy; General Surgery Department, Ospedale Maggiore, Parma, Italy; Denver Health Medical Center, Denver, Colorado USA; Virginia Commonwealth University, Richmond, Virginia USA; General Surgery, Macerata Hospital, Macerata, Italy; Department of Abdominal Surgery, University of Helsinki, Helsinki, Finland; General, Emergency and Trauma surgery, Maggiore Hospital, Bologna, Italy; Campinas Medical School, Campinas University, Campinas, Brazil

**Keywords:** Register, Open abdomen, Peritonitis, Pancreatitis, Trauma, Management, Surgery

## Abstract

Actually the most common indications for Open Abdomen (OA) are trauma, abdominal sepsis, severe acute pancreatitis and more in general all those situations in which an intra-abdominal hypertension condition is present, in order to prevent the development of an abdominal compartment syndrome. The mortality and morbidity rate in patients undergone to OA procedures is still high. At present many studies have been published about the OA management and the progresses in survival rate of critically ill trauma and septic surgical patients. However several issues are still unclear and need more extensive studies. The definitions of indications, applications and methods to close the OA are still matter of debate. To overcome this lack of high level of evidence data about the OA indications, management, definitive closure and follow-up, the World Society of Emergency Surgery (WSES) promoted the International Register of Open Abdomen (IROA). The register will be held on a web platform (Clinical Registers®) through a dedicated web site: www.clinicalregisters.org. This will allow to all surgeons and physicians to participate from all around the world only by having a computer and a web connection. The IROA protocol has been approved by the coordinating center Ethical Committee (Papa Giovanni XXIII hospital, Bergamo, Italy). IROA has also been registered to ClinicalTrials.gov (ClinicalTrials.gov Identifier: NCT02382770).

## Introduction

The Open Abdomen (OA) was firstly described almost 120 years ago by Andrew J. McCosh [1]. No popularity was gained by this technique in treating several severe conditions before it has been applied extensively to the severely injured patients in a damage control surgical strategy. Actually the most common indications for OA are trauma, abdominal sepsis, severe acute pancreatitis and more in general all those situations in which an intra-abdominal hypertension condition is present, in order to prevent the development of an abdominal compartment syndrome (ACS) [2, 3]. The mortality rates in patients underwent to OA are high, usually over 30 % depending on the patient cohort and on OA causative event [4]. The OA management is a complex and challenging situation that requires a multidisciplinary approach. In fact only by a close cooperation between surgeons and the ICU team would be possible to obtain good results in terms of survival improvement and morbidity reduction. In case of ACS in fact a therapy aiming to achieve early opening and early closure is the key. The “old” paradigm to “close at any cost” the abdomen shifted toward a combination of medical and surgical therapies including negative pressure wound therapy and dynamic closure, that would lead to a reduction in mortality, morbidity and incisional hernia rate.

At present many studies have been published about the OA management and the progresses in survival rate of critically ill trauma and septic surgical patients. All these results have only been obtained thanks to the great work of pioneers, scientific societies and their guidelines [5–9].

At present however several issues are still unclear and need more extensive studies. The definitions of indications, applications and methods to close the OA are still matter of debate. No definitive data demonstrated the real differences between the different techniques to maintain the OA in terms of morbidity and mortality. Patients treated with OA procedures are absolutely heterogeneous even within the same study. Large cohorts of patients treated with the same procedures are rare. Moreover no definitive data exist about nutrition strategies. Neither the impact of the different kind of nutrition on the outcomes has been defined [10–13]. All existing studies accrued patients in at least a few centers with many different biases [14–18]. Even few systematic review and meta-analysis have been published about the topic but no definitive data could be obtained [19, 20]. Lastly no sufficient data about the closure and follow-up of patients treated with OA strategies exist [21–23].

To overcome this lack of high level of evidence data about the OA indications, management, definitive closure and follow-up, the World Society of Emergency Surgery (WSES) promoted the International Register of Open Abdomen (IROA).

This prospective observational trial aims to enroll patients undergone to any kind of OA procedure.

**Fig. 1:**
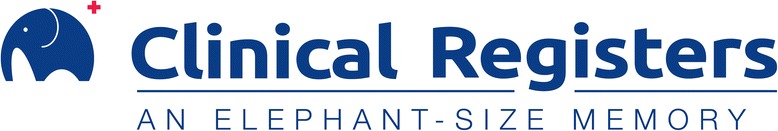
Clinical Register platform Logo

The web-based philosophy of the register will give the opportunity to all surgeons and physicians members of ICU teams treating with OA patients to participate. The register will be held on a web platform (Clinical Registers®) through a dedicated web site: www.clinicalregisters.org (Fig. 1). This will allow to all surgeons and physicians to participate from all around the world only by having a computer and a web connection.

The data insertion will be possible after registration to the web platform. Each surgeon will get personal credentials that will allow him/her to register patients. Data will be enrolled and kept protected by a certified system of data encryptation.

The IROA protocol has been approved by the coordinating center Ethical Committee (Papa Giovanni XXIII hospital, Bergamo, Italy). IROA has also been registered to ClinicalTrials.gov (ClinicalTrials.gov Identifier: NCT02382770). All necessary documents can be downloaded from the register web-site. A free access web-site part will allow to all those who may need more information, to obtain them without the necessity of registration. Each year will be published a paper containing the registered data with all the names of participating physicians. All physicians who enrolled patients can ask to have their own data according to the protocol rules.

WSES strongly believe in the necessity to diffuse emergency and trauma surgery as well as acute care surgery knowledge and to create diffuse collaboration in worldwide scientific projects. For this reason the present paper aims to warmly invite all surgeons or physicians who perform and manage with OA procedures to participate to this international effort in order to get the best result and contribute to better understand the OA procedure.
